# Supplementary figures and images for: Homoharringtonine Promotes FTO Degradation to Suppress LILRB4‐Mediated Immune Evasion in Acute Monocytic Leukaemia
Source: Cell Prolif. 2025 Jul 1;59(2):e70090. doi: 10.1111/cpr.70090 (PMC12877946; doi:10.1111/cpr.70090)

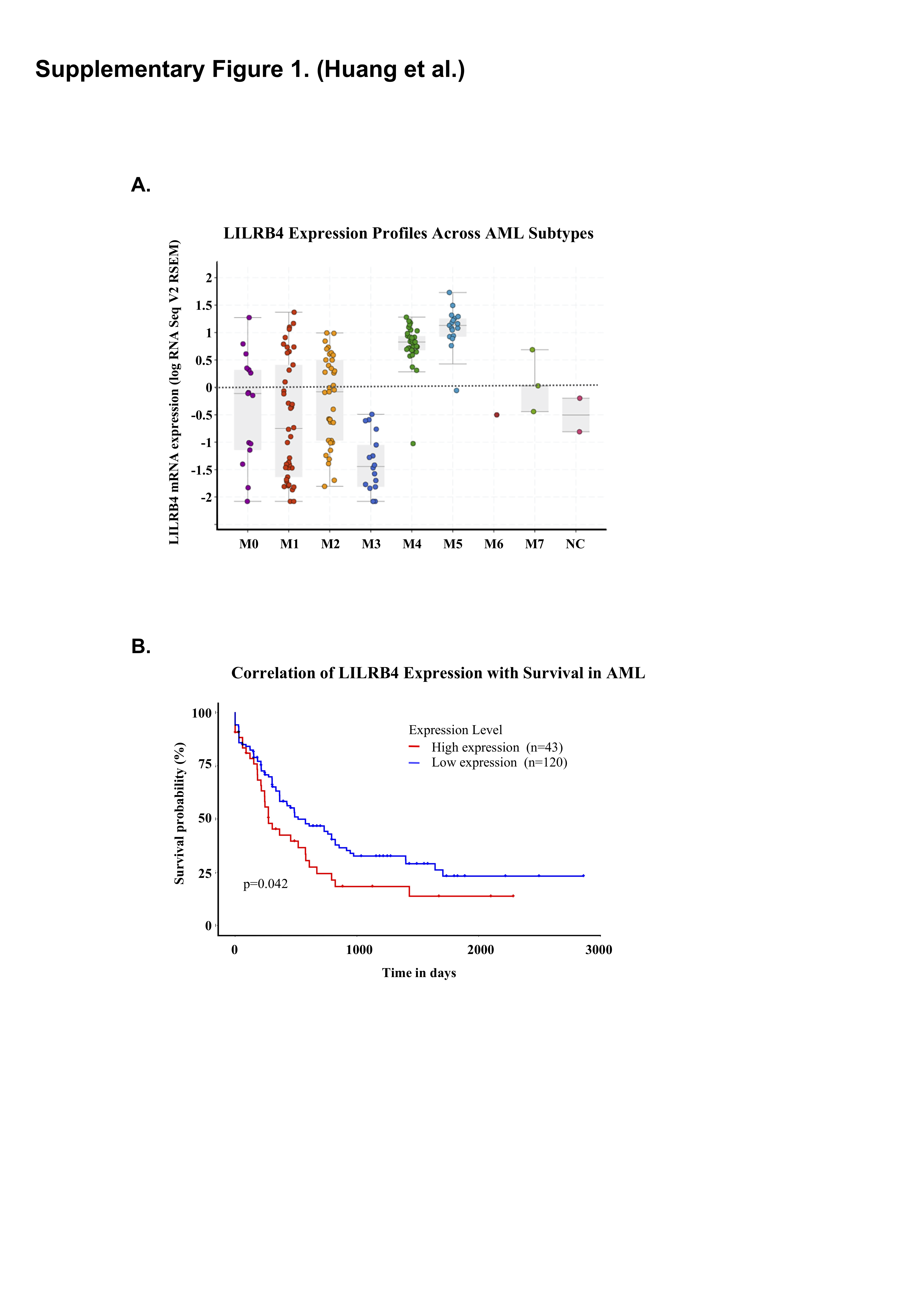

Supplement: Supplementary file 2 — Figure S1. LILRB4 Expression is Closely Associated with Clinical Patient Survival Rates. [file CPR-59-e70090-s006.png]

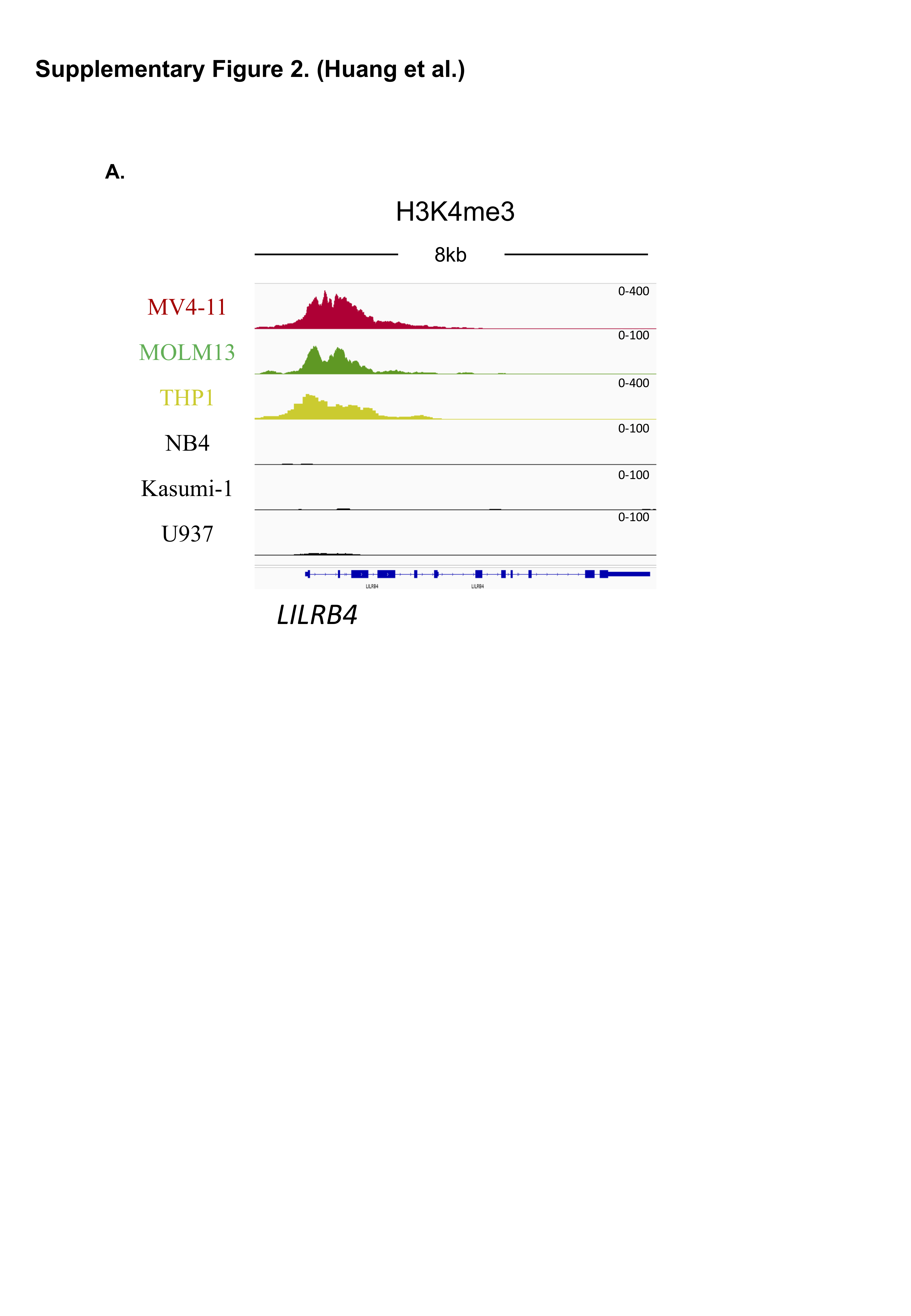

Supplement: Supplementary file 3 — Figure S2. Analysis of H3K4me3 Enrichment at LILRB4 Locus in AML Cell Lines. [file CPR-59-e70090-s002.png]

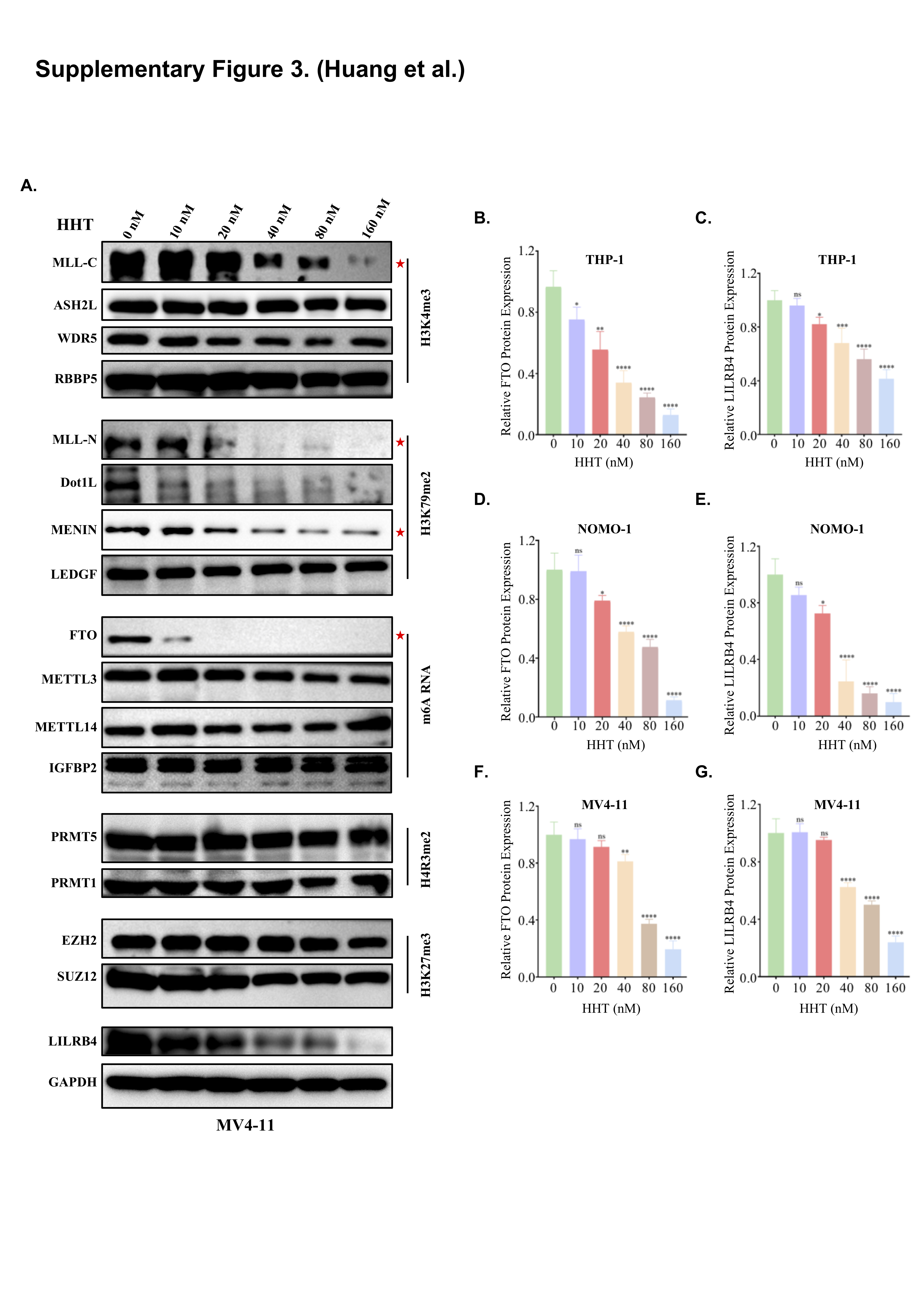

Supplement: Supplementary file 4 — Figure S3. HHT Modulates Epigenetic Regulators and Alters FTO and LILRB4 Expression in AML cells. [file CPR-59-e70090-s008.png]

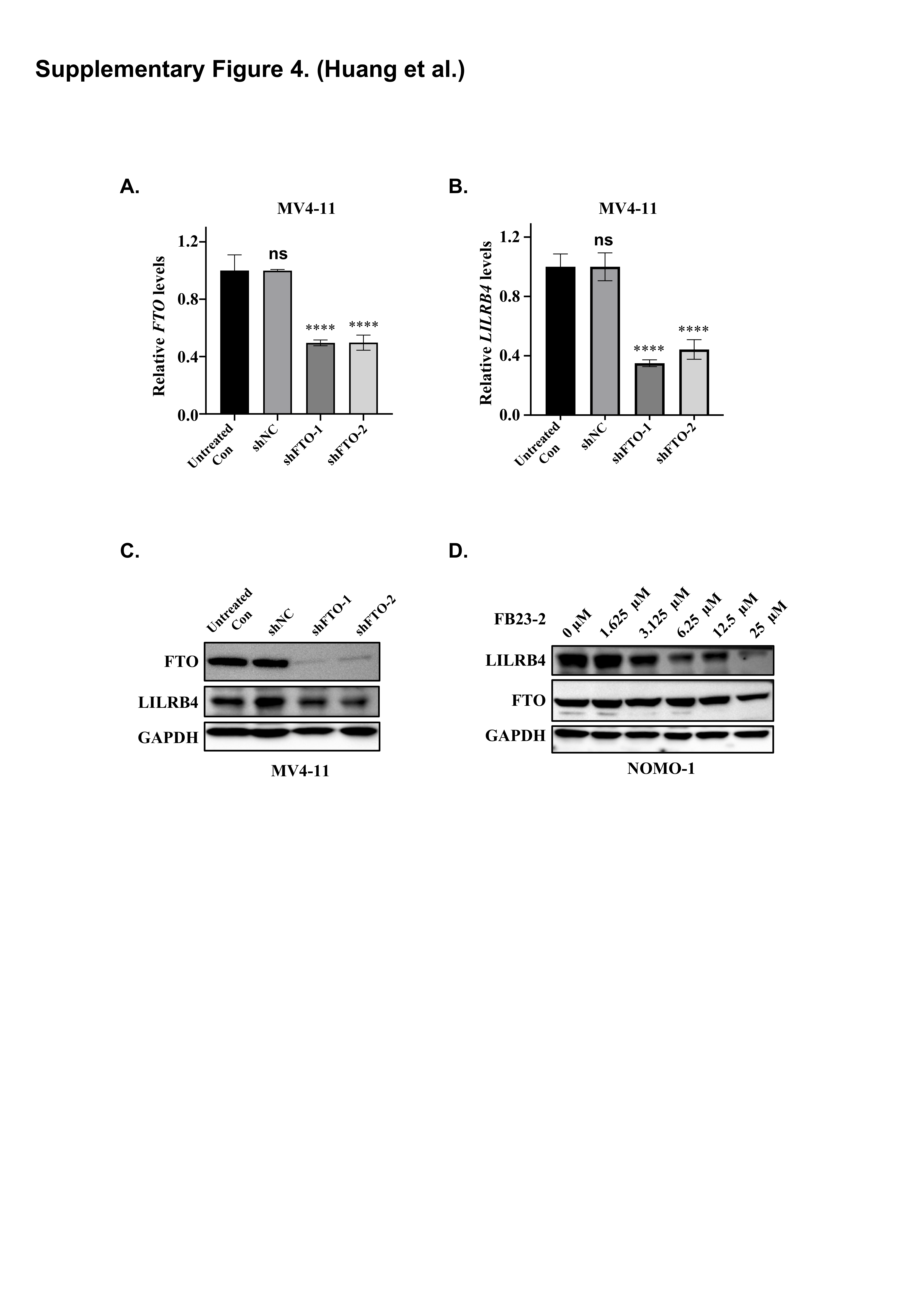

Supplement: Supplementary file 5 — Figure S4. FTO Regulates LILRB4 Expression Levels. [file CPR-59-e70090-s004.png]

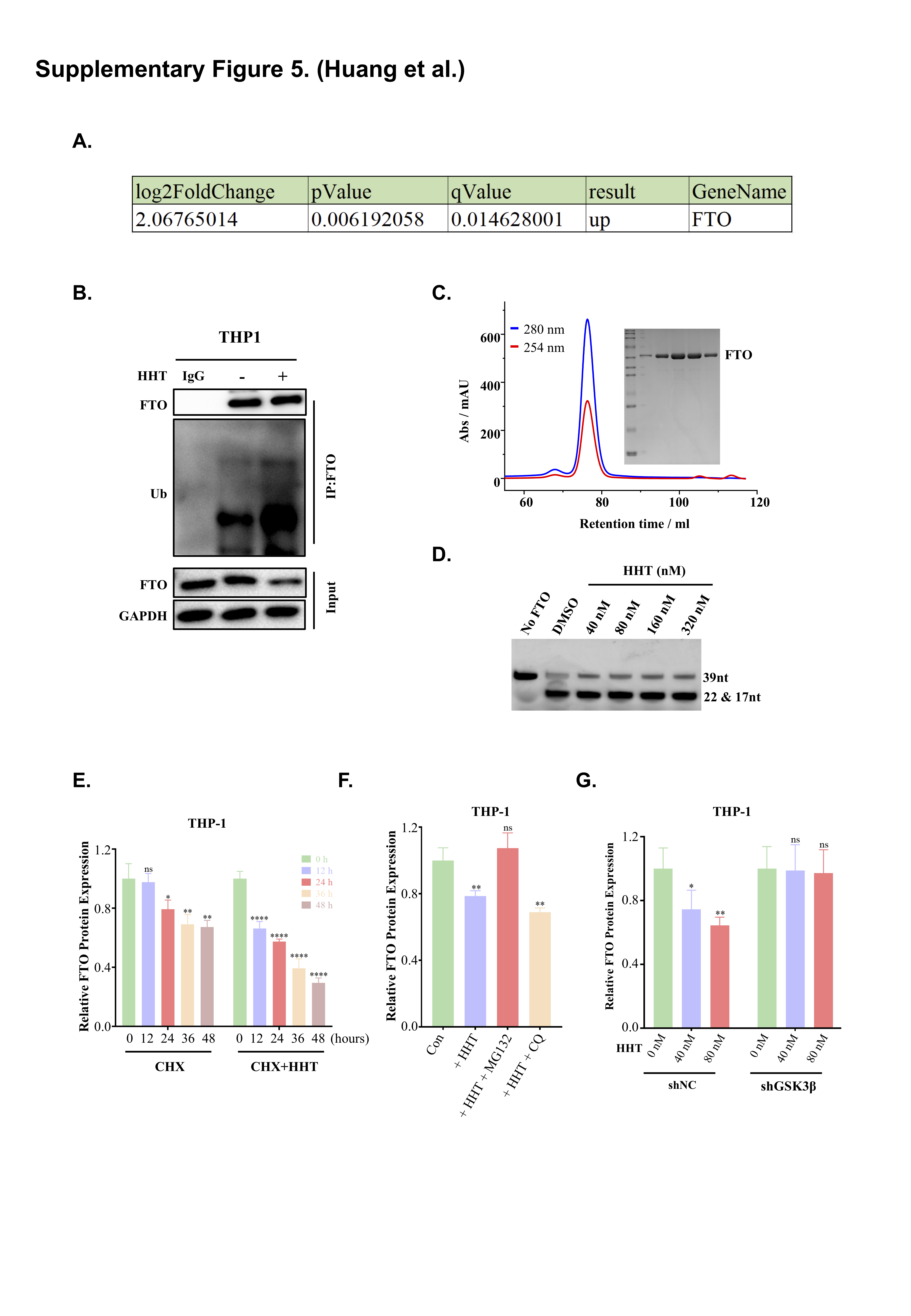

Supplement: Supplementary file 6 — Figure S5. HHT Promotes FTO Degradation via Ubiquitination to Suppress FTO Protein Expression. [file CPR-59-e70090-s001.png]

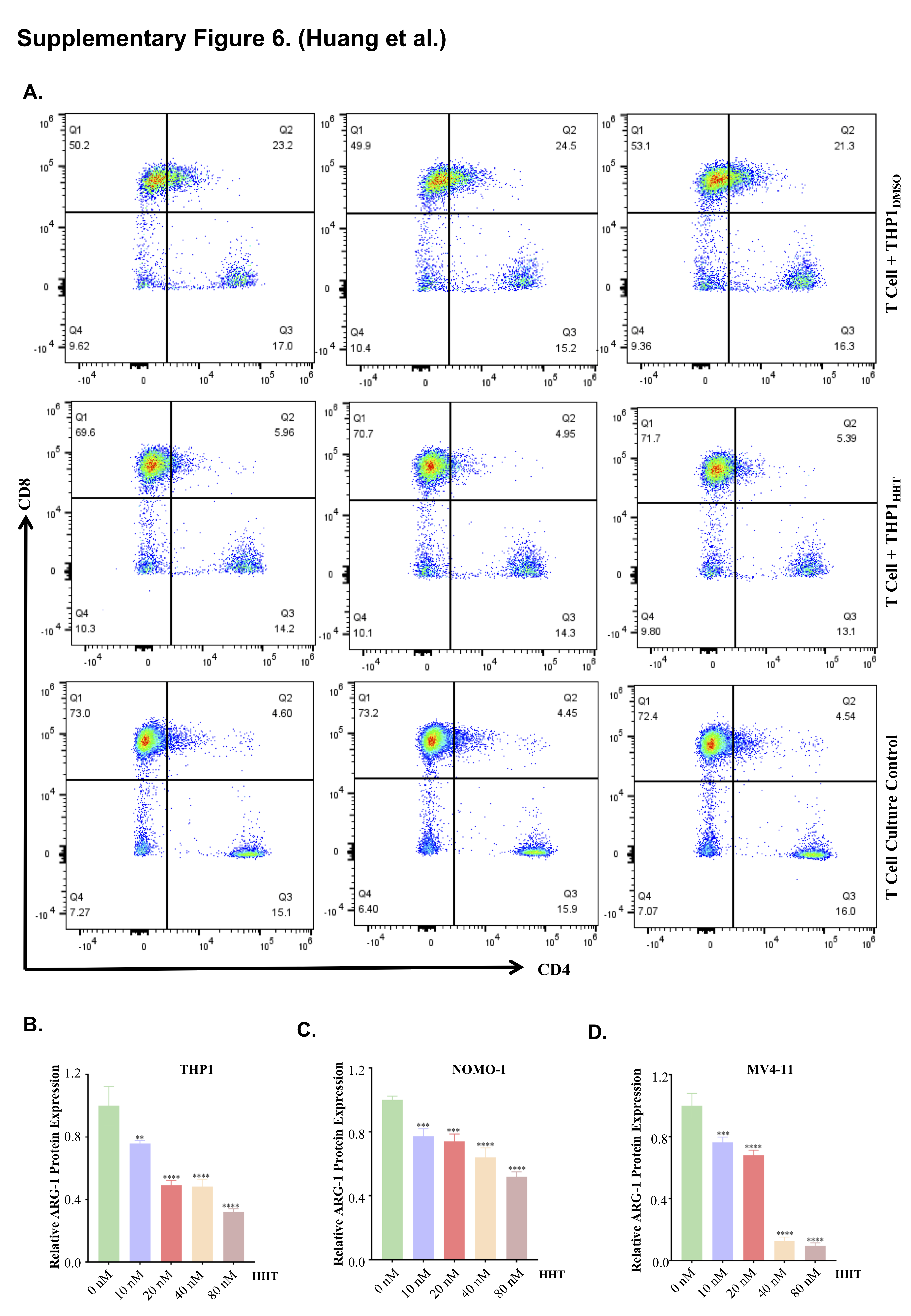

Supplement: Supplementary file 7 — Figure S6. HHT reduces AML‐mediated T cell suppression and downregulates ARG‐1 in AML cells. [file CPR-59-e70090-s003.png]

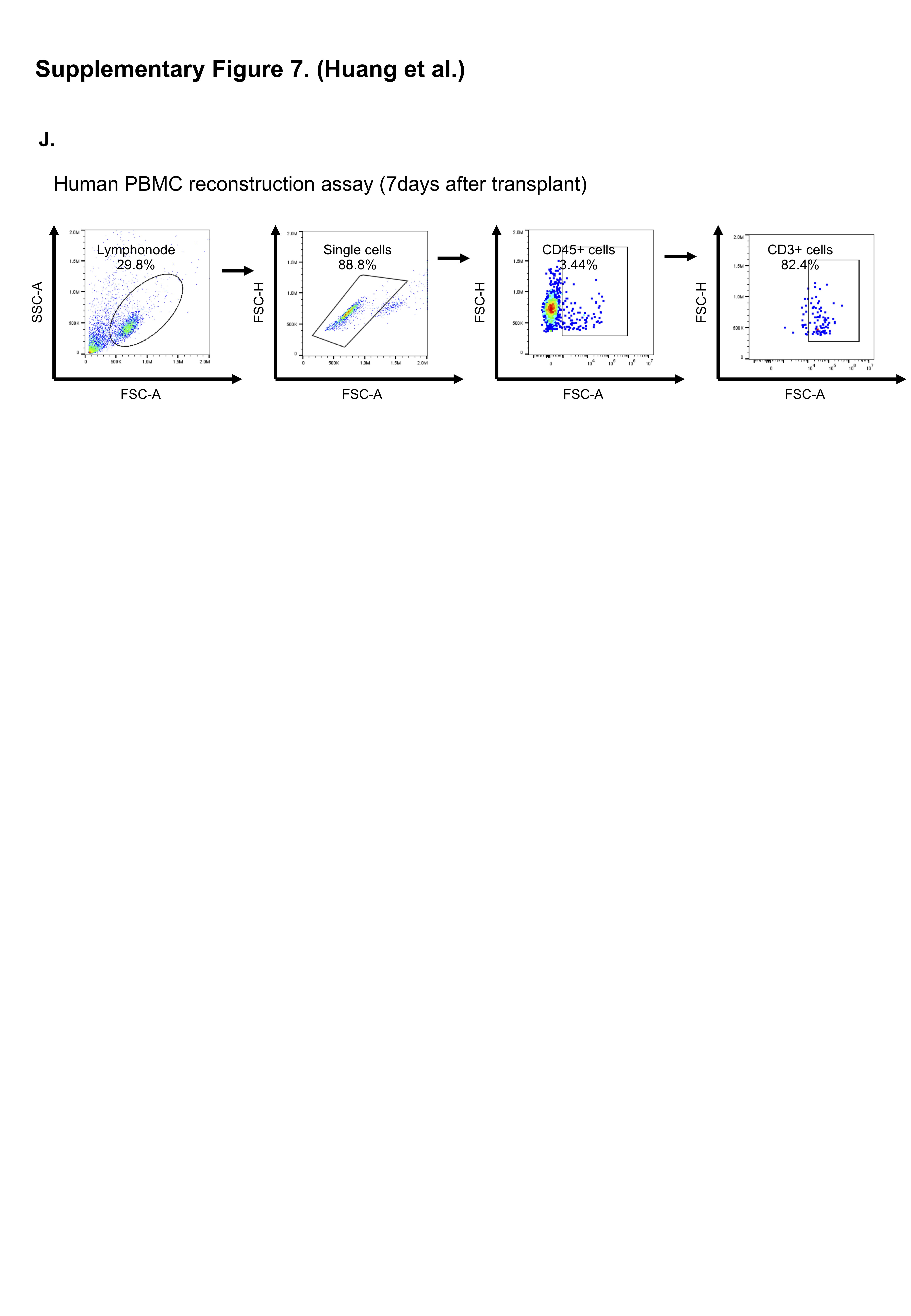

Supplement: Supplementary file 8 — Figure S7. HHT Inhibits Immunevasion in Humanised Mouse AML Xenografts. [file CPR-59-e70090-s005.zip › cpr70090-sup-0005-FigureS2-S7@Supplementary Figure S7-2.png]

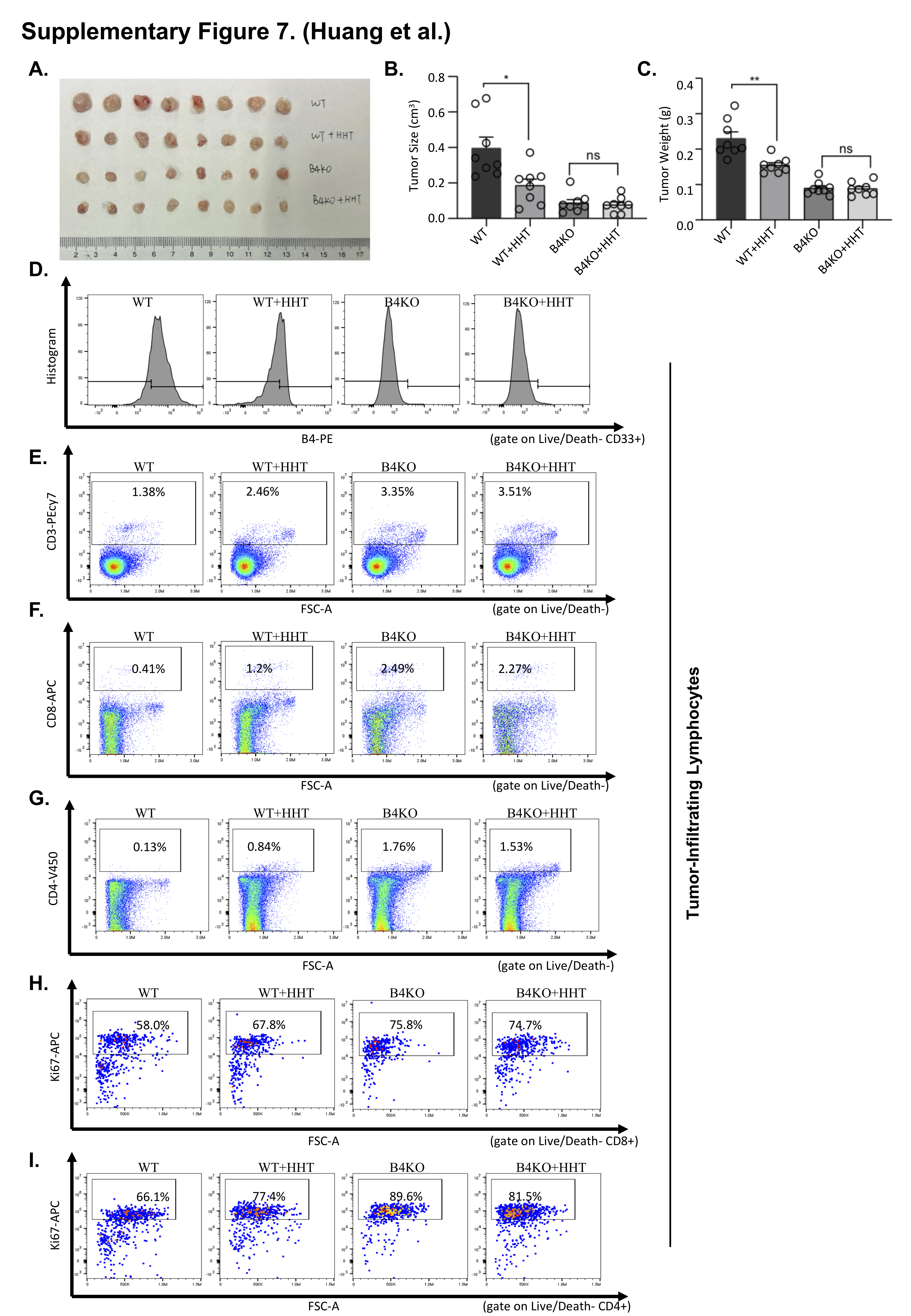

Supplement: Supplementary file 8 — Figure S7. HHT Inhibits Immunevasion in Humanised Mouse AML Xenografts. [file CPR-59-e70090-s005.zip › cpr70090-sup-0003-FigureS1-S7@Supplementary Figure S7-1.png]

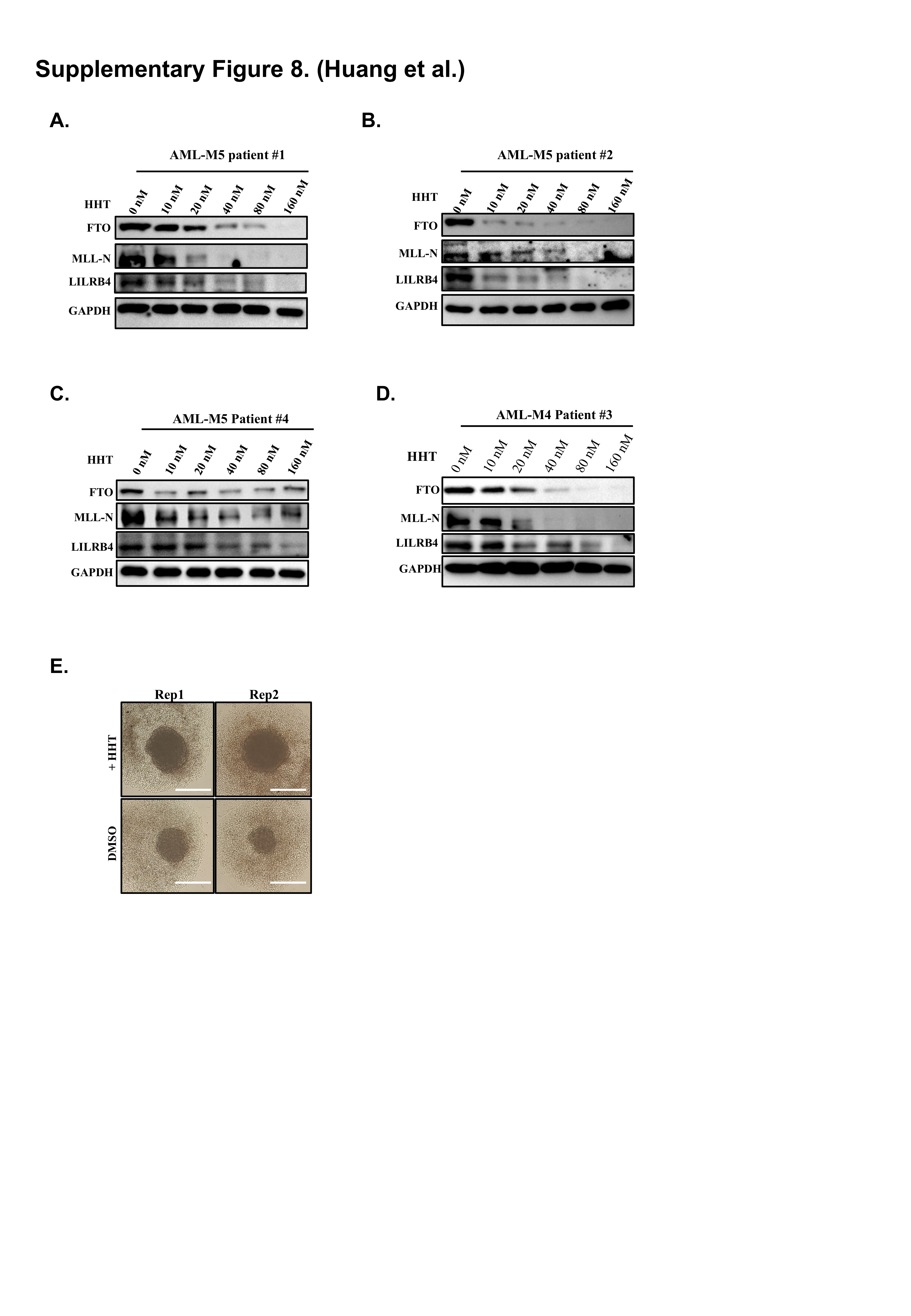

Supplement: Supplementary file 9 — Figure S8. HHT Suppresses LILRB4 Expression in Primary Cells from AML‐M4/5 Clinical Samples. [file CPR-59-e70090-s007.png]
